# A Unified Flash Memory Platform for Mode‐Adaptive and Robust AI Computation

**DOI:** 10.1002/advs.76619

**Published:** 2026-07-21

**Authors:** Dayeon Yu, Hwiho Hwang, Byeongchan Oh, Junmo Lee, Shimeng Yu, Tae‐Hyeon Kim, Hyungjin Kim

**Affiliations:** ^1^ Division of Materials Science and Engineering and Department of Semiconductor Engineering Hanyang University Seoul South Korea; ^2^ Department of Semiconductor Engineering Seoul National University of Science and Technology Seoul South Korea; ^3^ School of Electrical and Computer Engineering Georgia Institute of Technology Atlanta Georgia USA

**Keywords:** AND‐type array, compute‐in‐memory (CIM), dual‐mode operation, flash memory, hardware‐based neural network

## Abstract

Computing‐in‐memory (CIM) architectures offer a promising route toward energy‐efficient artificial intelligence by reducing data‐movement overhead. However, most existing CIM hardware operates at a fixed trade‐off between accuracy, energy efficiency, and robustness, limiting adaptability to diverse workloads. Here, we present a dual‐mode CIM accelerator based on an AND‐type charge‐trap flash array that enables energy‐adaptive operation without device‐level structural modification. By integrating transistor‐mode current sensing and capacitor‐mode charge sensing in the same device structure, the proposed architecture allows flexible switching between high‐precision computation and ultra‐low‐power, noise‐resilient operation within a single hardware platform through peripheral switching associated with each sensing mode. Experimental results demonstrate reliable vector–matrix multiplication, hardware neural network inference, and strong tolerance to device and voltage variations. System‐level benchmarking further confirms improved energy efficiency and reduced peripheral overhead. This work establishes a practical and scalable CIM platform that dynamically balances performance and robustness, providing a versatile foundation for energy‐adaptive artificial intelligence (AI) hardware.

## Graphical abstract

A unified AND‐type flash memory platform enables both transistor‐mode and capacitor‐mode computing‐in‐memory operations within the same device structure. By selectively switching the sensing mode through peripheral reconfiguration, the platform provides adaptable trade‐offs between computational accuracy, robustness, and energy efficiency for AI inference workloads.

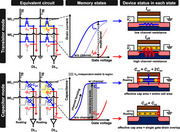

## 1 Introduction

The rapid expansion of artificial intelligence (AI) has led to an unprecedented increase in computational demand, exposing the fundamental energy and latency limitations associated with data movement between memory and processing units, commonly referred to as the memory wall [1, 2, 3, 4, 5, 6, 7, 8, 9]. As AI models continue to scale in size and complexity, particularly in edge and energy‐constrained environments, improving energy efficiency has become as critical as achieving high computational accuracy. Computing‐in‐memory (CIM) architectures have therefore emerged as a promising paradigm by enabling computation directly within memory arrays, significantly reducing data transfer overhead and energy consumption [10, 11, 12, 13, 14, 15, 16, 17, 18]. Despite these advantages, most existing CIM accelerators operate at a fixed design point in the trade‐off space between computational accuracy, energy efficiency, and reliability. In practice, however, AI workloads exhibit highly diverse and dynamic requirements. Training, calibration, and accuracy‐critical inference demand high precision and throughput, whereas edge inference, always‐on sensing, and long‐term operation prioritize ultra‐low power consumption and robustness against device variability, noise, and aging effects [19, 20, 21, 22, 23, 24, 25, 26, 27]. The inability of current CIM hardware to adapt to such heterogeneous workload demands fundamentally limits its energy efficiency and practical applicability at the system level.

Charge‐trap flash (CTF) memory has been widely explored as a CIM platform thanks to its mature fabrication process, excellent retention characteristics, and scalability [28, 29, 30, 31, 32, 33, 34, 35, 36]. Conventional NAND flash‐based CIM architectures, however, suffer from intrinsic limitations arising from their serial string configuration. As the string length increases, the available current decreases and sensing margins degrade, while access latency and peripheral overhead grow correspondingly [37, 38, 39, 40, 41, 42, 43, 44, 45, 46]. These architectural constraints restrict parallelism in vector–matrix multiplication (VMM) and introduce additional energy overhead at both the array and circuit levels, particularly in large‐scale implementations. To overcome these limitations, AND‐type flash arrays with inherently parallel structures have been proposed [47, 48, 49, 50, 51, 52, 53, 54, 55, 56, 57, 58, 59, 60, 61, 62]. In AND‐type architectures, memory cells controlled by independent wordlines (WLs) are connected in parallel along shared drain lines, enabling efficient current summation and making them well suited for CIM operations. From an architectural perspective, this parallelism offers clear advantages in both throughput and energy efficiency. Nevertheless, most flash‐based CIM implementations, including those based on AND‐type arrays, rely exclusively on a single sensing modality: either transistor‐mode (current‐based) sensing or capacitor‐mode (charge‐based) sensing.

Transistor‐mode sensing supports multilevel weight representation and fast readout, making it suitable for performance‐critical workloads. However, it typically incurs higher dynamic energy consumption and increased sensitivity to device‐level variations, read noise, and analog‐to‐digital conversion (ADC) overhead [63]. In contrast, capacitor‐mode sensing enables binary operation with near‐zero static power consumption and strong tolerance to noise and variability, yet its limited precision and slower sensing dynamics restrict its use in applications requiring high inference accuracy [64, 65, 66, 67, 68, 69, 70, 71]. As a result, most current CIM accelerators are confined to a narrow operating regime, forcing designers to choose between energy‐efficient but low‐precision hardware and high‐accuracy but energy‐intensive solutions. From an energy‐systems perspective, this fixed operating point can be a critical bottleneck. System‐level AI implementations would benefit substantially from hardware that can dynamically adapt its operating mode, prioritizing accuracy when power is abundant and switching to robust, ultra‐low‐power operation under energy‐constrained or noisy conditions. However, enabling such adaptability within a single CIM platform remains an open challenge, as most prior approaches require separate device structures, additional peripheral circuits, or fundamentally different memory technologies.

In this work, we address this challenge by presenting a dual‐mode CIM accelerator based on an AND‐type CTF array that integrates both transistor‐mode and capacitor‐mode sensing within the same TiN/Al_2_O_3_/Si_3_N_4_/SiO_2_/poly‐Si (TANOS) device stack. Since both sensing schemes are implemented using the same CTF memory cell structure and array stack, the proposed architecture supports flexible selection of the sensing mode without requiring device‐level structural modification, while the mode selection is achieved through the corresponding bias and readout conditions. Transistor‐mode provides multilevel, high‐throughput computation for accuracy‐critical tasks, while capacitor‐mode offers binary, charge‐based operation with near‐zero static power consumption and strong robustness against device and voltage variations. By enabling flexible operation across the energy–accuracy–reliability design space, the proposed dual‐mode architecture expands the energy‐efficiency envelope of flash‐based CIM. Through comprehensive evaluation across device, array, and system levels, we demonstrate that the proposed platform can flexibly balance computational accuracy and energy efficiency through workload‐dependent sensing‐mode selection. This energy‐adaptive CIM approach provides a practical route toward workload‐adaptive and energy‐aware AI hardware.

## 2 Device Structure and Operating Principles

**FIGURE 1:**
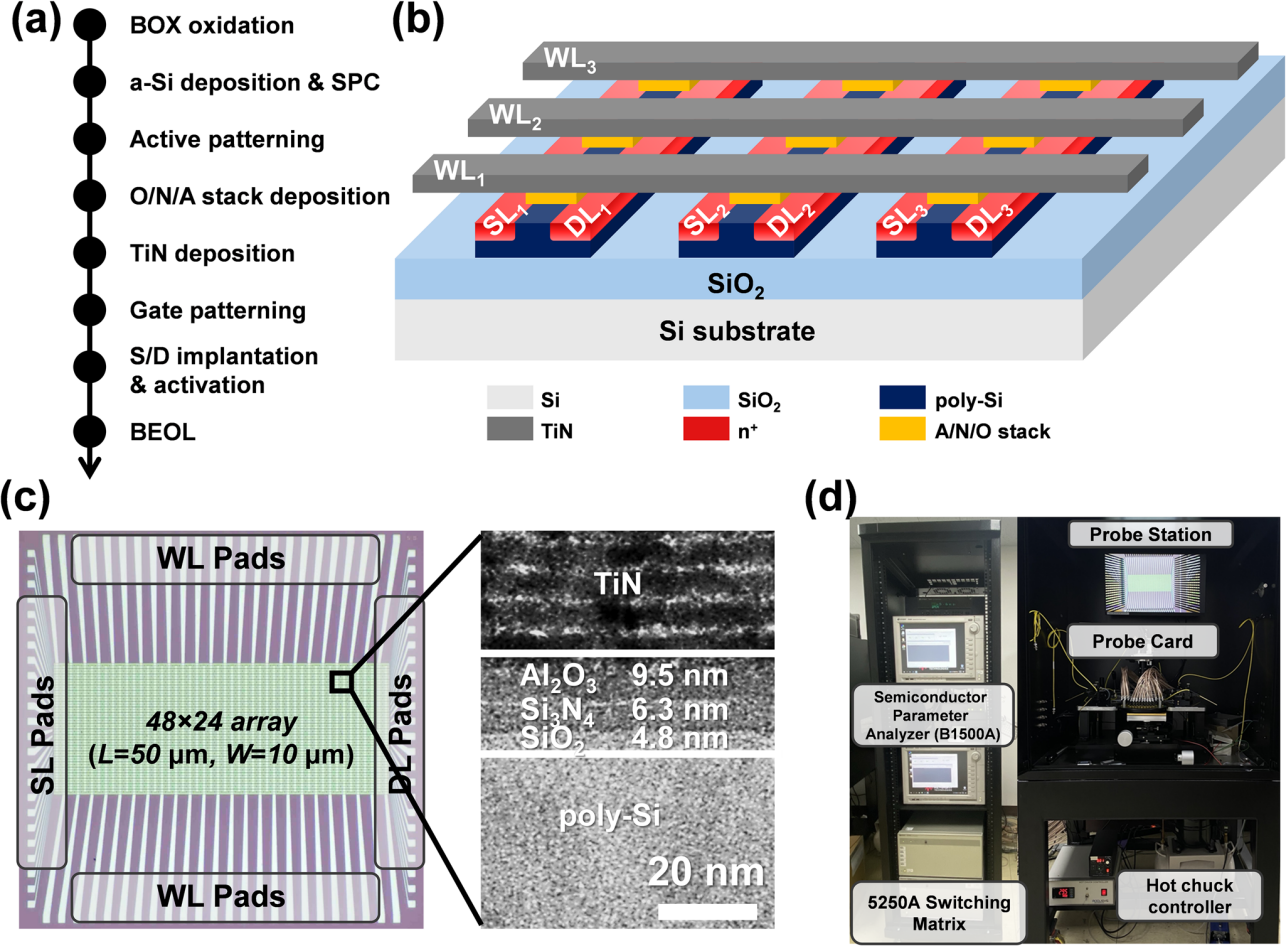
AND‐type flash array architecture and experimental setup. (a) Fabrication process flow of the AND flash memory cell with a TiN/Al_2_O_3_/Si_3_N_4_/SiO_2_/poly‐Si (TANOS) charge‐trap stack. (b) Schematic illustration of the AND‐type array architecture, where memory cells controlled by independently addressed WLs are connected in parallel along shared SLs and DLs, enabling current summation for VMM operations. (c) Top‐view optical microscope image of the fabricated 48 × 24 AND flash array with a cross‐sectional transmission electron microscopy (TEM) image showing the TANOS gate stack. (d) Electrical measurement setup used for transistor‐ and capacitor‐mode characterizations.

The AND‐type CTF array with a TANOS stack was fabricated following the process flow illustrated in Figure 1a. A 300 nm buried oxide (BOX) layer was first formed on a p‐type (100) silicon wafer by furnace wet oxidation. Subsequently, a 100 nm amorphous silicon layer was deposited on the BOX using low‐pressure chemical vapor deposition (LPCVD) at 550°C with a SiH_4_ flow rate of 100 sccm, followed by solid‐phase crystallization (SPC) at 600°C for 24 h. SPC was employed to obtain a poly‐Si channel with uniform electrical characteristics suitable for array‐level operation. The active regions and source lines (SLs) and drain lines (DLs) were then defined by photolithography and patterning. A 4.8 nm SiO_2_ tunneling oxide was deposited by LPCVD at 800°C using dichlorosilane (DCS, 40 sccm) and N_2_O (80 sccm), followed by deposition of a 6.3 nm Si_3_N_4_ charge‐trap layer at 800°C using DCS (40 sccm) and NH_3_ (400 sccm). A 9.5 nm Al_2_O_3_ blocking oxide was subsequently deposited by atomic layer deposition (ALD) at 340°C using trimethylaluminum (TMA) as the precursor. A 50 nm TiN gate electrode was then deposited by metal–organic chemical vapor deposition (MOCVD). Prior to gate patterning, a 15 nm SiO_2_ hard mask was deposited by plasma‐enhanced chemical vapor deposition (PECVD) at 380°C to improve adhesion between the TiN layer and the photoresist during subsequent etching processes. After photolithography, the SiO_2_ hard mask was etched using buffered oxide etchant (BOE, HF:NH_4_F = 1:100) at room temperature. TiN gate patterning was performed by inductively coupled plasma (ICP) etching followed by wet etching in diluted hydrogen peroxide (H_2_O_2_:DI = 1:4) at 67°C. The source and drain regions were formed by As^+^ ion implantation at a dose of 2 × 10^15^ cm^−2^ and an energy of 40 keV, followed by dopant activation via rapid thermal annealing (RTA). Finally, a 300 nm interlayer dielectric (ILD) was deposited by PECVD, and contact‐hole patterning and metallization were carried out to complete the back‐end‐of‐line (BEOL) process. All fabrication steps were carried out using CMOS‐compatible processes, ensuring scalability and compatibility with conventional flash memory manufacturing.

The array structure, in which devices controlled by independently addressed wordlines (WLs) are connected in parallel along shared source lines (SLs) and drain lines (DLs), is illustrated in Figure 1b. This parallel connectivity enables simultaneous current summation, making the array well suited for efficient VMM operations required for CIM applications. Figure 1c presents a top‐view optical microscope image of the fabricated 48 × 24 AND‐type flash array, together with a cross‐sectional transmission electron microscopy (TEM) image confirming the formation of the TANOS stack. To verify dual‐mode operations and evaluate the electrical characteristics of both transistor‐mode and capacitor‐mode sensing, the fabricated array was characterized with the measurement system, as shown in Figure 1d. The array was interfaced with a semiconductor parameter analyzer and a switching matrix, and all measurements were controlled using a custom‐developed C++ control software. The detailed characterization methods are explained in the Experimental Section.

**FIGURE 2:**
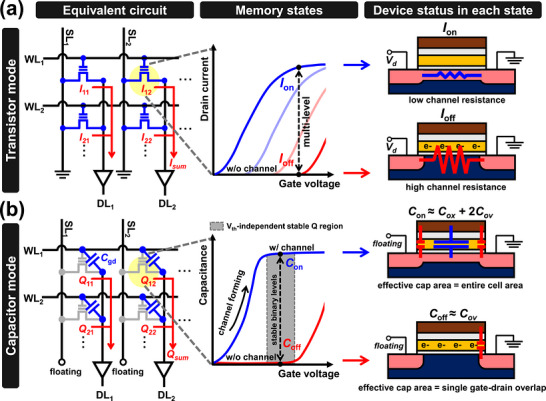
Dual‐mode operation mechanisms of AND‐type flash memory cell. (a) Transistor‐mode sensing scheme, where SL is grounded, and a read voltage is applied to DL to sense the device state through the resulting drain current. Multilevel states are realized by modulating channel formation, enabling current‐based weight representation for high‐precision computation. (b) Capacitor‐mode sensing scheme, where the SL is electrically isolated and left floating, and the device state is sensed through the effective capacitance between WL and DL. Before channel formation, only the gate‐to‐drain *C*_ov_ contributes, yielding a low‐capacitance state, *C*_off_, while channel formation above *V*_th_ introduces additional capacitive coupling, resulting in a high‐capacitance state, *C*_on_.

Although the AND‐type flash array is fundamentally based on a transistor structure, it supports both transistor‐mode and capacitor‐mode sensing through different readout schemes, as illustrated in Figure 2a,b, respectively. In transistor‐mode, the SL is grounded, and a read voltage is applied to the DL, allowing the device state to be sensed through the resulting drain current. Multi‐level states are realized by modulating channel formation, effectively tuning the device conductance to generate distinct current levels within the on–off window. Owing to its current‐based sensing mechanism and fast response, transistor‐mode operation is well suited for performance‐ and accuracy‐critical applications. By contrast, in capacitor‐mode, the SL is electrically isolated from ground and left floating, and the device state is sensed through the effective capacitance formed between the WL and DL. The SL is intentionally left floating during capacitor‐mode operation, enabling mode selection through a simple SL switching scheme without requiring additional bias circuitry. Before channel formation, only the gate‐to‐drain overlap capacitance (*C*_ov_​) contributes to the total capacitance, resulting in a low‐capacitance state (*C*_off_​). As the gate voltage increases and channel formation begins, the effective capacitive coupling between the WL and DL expands. Once the gate voltage exceeds the threshold voltage (*V*_th_​), additional contributions from the source‐side *C*_ov_​ and the gate oxide capacitance (*C*_ox_​) are added, yielding a high‐capacitance state (*C*_on_​) of approximately 2*C*_ov_ + *C*_ox_​. Because capacitor‐mode operates with only two well‐separated states, it provides strong robustness against device‐to‐device variation and external noise. Moreover, since the readout relies on charge sensing through capacitive coupling, the static power consumption is negligible. In addition, the simplified binary sensing in capacitor‐mode reduces the burden on peripheral readout circuits, contributing to improved system‐level energy efficiency. These characteristics make capacitor‐mode operation particularly suitable for energy‐constrained and long‐term stable computing‐in‐memory applications. Together, the two sensing modes provide complementary operating characteristics, enabling flexible trade‐offs between computational performance and energy efficiency within the same AND‐type array platform through peripheral reconfiguration associated with each sensing mode.

## 3 Transistor‐ and Capacitor‐Mode Operations

**FIGURE 3:**
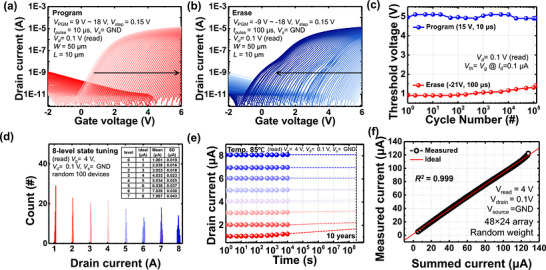
Program/erase characteristics and transistor‐mode VMM operation of AND flash array. (a) Transfer characteristics of the AND‐type flash memory cell during ISPP, showing a gradual rightward shift of the threshold voltage *V*_th_ with increasing program pulse amplitude. (b) Transfer characteristics during ISPE, where successive erase pulses induce a leftward shift of *V*_th_​, returning the device toward its initial state. (c) Endurance characteristics of the device, demonstrating stable program and erase operation over 10^5^ cycles. (d) Histogram of eight programmed current levels obtained from 100 randomly selected devices in the array, showing the mean and standard deviation for each target level and confirming precise multi‐level current tuning. (e) Retention characteristics of the eight programmed current levels measured at 85°C, indicating stable retention and clear level separation extrapolated to long‐term operation. (f) Experimental verification of analog VMM operation in a 48 × 24 array, where the measured DL current exhibits a strong linear correlation with the arithmetic sum of individual device currents.

Figure 3a shows the transfer characteristics of the device during programming based on Fowler–Nordheim (FN) tunneling. Incremental step pulse programming (ISPP) [72] was performed by applying 10 µs gate pulses from 9 to 18 V in 0.15 V increments while grounding both the source and drain. As the ISPP voltage increased, *V*_th_​ shifted toward higher values, resulting in a memory window of over 6 V. Conversely, Figure 3b presents the erase characteristics. An initial 18 V, 10 µs program pulse was followed by incremental step pulse erase (ISPE) voltages ranging from −9 to −18 V in −0.15 V increments, with a pulse width of 100 µs applied to the gate. This process induced a leftward shift of *V*_th_, eventually restoring the device to a state close to its initial condition. Through these program and erase operations, multiple current levels can be generated at a fixed read voltage, enabling multilevel storage in transistor‐mode.

Figure 3c shows the endurance characteristics of the device, demonstrating stable program and erase operation over 10^5^ cycles using 15 V, 10 µs program pulses and −21 V, 100 µs erase pulses. Based on this stable write‐and‐verify scheme, 100 randomly selected devices within a single array were individually tuned to eight distinct current levels ranging from 1 to 8 µA in 1 µA increments, using the bias conditions illustrated in Figure S1a. The resulting current distributions are summarized as histograms in Figure 3d, with the corresponding mean and standard deviation for each target level. All programmed levels exhibit a mean absolute error of approximately 0.1 µA, demonstrating highly precise and reproducible current‐level tuning across the array. During the programming or erasing of a selected cell, the program or erase voltage was applied to the corresponding WL, while the surrounding eight neighboring cells were biased using a *V*/2 inhibit scheme. As confirmed in Figure S1b,c, the unselected neighboring cells maintained their original *V*_th_ without noticeable disturbance, verifying effective inhibit operation within the AND‐type array. With this write‐and‐verify scheme, a 48 × 24 down‐sampled Cameraman image encoded in 3‐bit resolution was selected as a target pattern, as shown in Figure S2a. Each device in the 48 × 24 AND‐type array was programmed to one of eight discrete current levels corresponding to the target image, and the resulting drain current map was subsequently measured. The resulting map closely reproduces the target image, demonstrating accurate and uniform weight transfer in the AND‐type array under current‐based transistor‐mode operation. This result demonstrates that the AND‐type flash array can reliably support multi‐level weight programming for large‐scale analog CIM applications.

Also, the retention characteristics were evaluated by monitoring these eight programmed levels for 10^4^ s at 85°C, as shown in Figure 3e. Extrapolation of the measured data indicates that all eight levels remain clearly distinguishable for up to 10 years (3 × 10^8^ s), confirming excellent charge retention. Such stable endurance and retention characteristics are essential for maintaining weight accuracy and minimizing recalibration overhead in energy‐efficient CIM systems. Finally, Figure 3f evaluates the analog VMM capability of the array. After randomly programming device states within a 48 × 24 array, the number of activated WLs was gradually increased, and the measured DL current was compared with the arithmetic sum of individual device currents. The resulting correlation exhibits an *R* ^2^ value of 0.999, confirming highly accurate analog current summation suitable for VMM operations.

**FIGURE 4:**
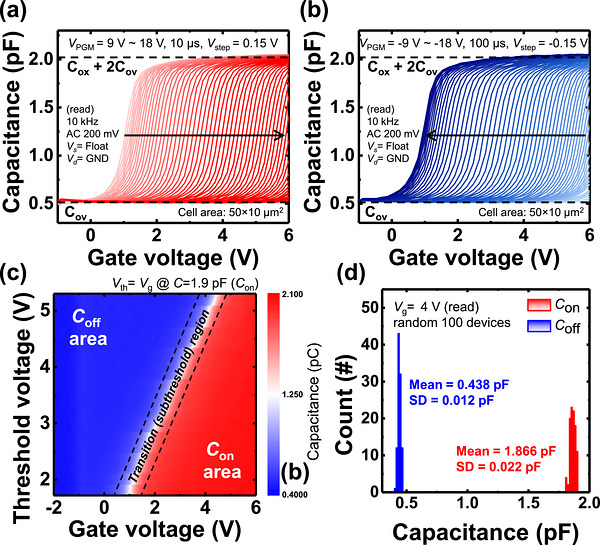
Capacitor‐mode sensing characteristics. (a) *C–V* characteristics of the AND‐type flash memory cell measured in capacitor‐mode under ISPP operation, showing the transition from *C*_off_ to *C*_on_ as channel formation occurs above *V*_th_. (b) Erase characteristics of the device in capacitor‐mode, where ISPE induces a negative shift of *V*_th_​, modulating the device capacitance state. (c) Extracted device capacitance as a function of gate voltage for different programmed states, illustrating stable capacitance plateaus outside the transition region and enabling robust binary state sensing. (d) Histograms of *C*_on​_ and *C*_off_​ extracted from 100 randomly selected devices in the array, demonstrating tight distributions with small standard deviation and clear binary separation across the array.

Figure 4a presents the *C–V* characteristics of a device with a channel width of 50 µm and a channel length of 10 µm measured in capacitor‐mode, where the SL is left floating and the capacitance between the WL and DL is sensed using a 10 kHz, 200 mV small‐signal AC voltage. During the ISPP, 10 µs gate pulses ranging from 9 to 18 V in 0.15 V increments shift *V*_th_ toward higher values. When the gate voltage is below *V*_th_, only the gate‐to‐drain *C*_ov_ contributes to the total capacitance, resulting in *C*_off_​. Once the gate voltage exceeds *V*_th_​, channel formation enhances the capacitive coupling, and additional contributions from the source‐side overlap capacitance *C*_ov_​ and *C*_ox_ increase the total capacitance to *C*_on_, approximately equal to 2*C*_ov_ + *C*_ox_. Figure 4b shows the erase characteristics obtained by applying an initial 18 V, 10 µs program pulse followed by the ISPE pulses ranging from −9 to −18 V in 0.15 V increments with a pulse width of 100 µs, resulting in a negative shift of *V*_th_​. Although the measured capacitance varies with both the device state and the applied gate voltage, the device consistently outputs either *C*_off_ (≈ 0.5 pF) or *C*_on_ (≈ 2.0 pF) outside the transition region, maintaining clear binary separation.

From the measured *C–V* characteristics depending on the device state, *V*_th_ of each device was extracted by defining *V*_th_ as the gate voltage at which *C*_on_ is consistently observed, and the device capacitance value is plotted according to the gate voltage, as shown in Figure 4c. These results confirm that, for a given device, the capacitance remains stable outside the transition region: *C*_off_ is consistently observed at low gate voltages, while *C*_on_​ is obtained at gate voltages well above *V*_th_. This wide separation enables robust state sensing outside the transition region. Similar behavior can be observed when the gate voltage is fixed. The devices with higher *V*_th_ ​ reliably maintain the *C*_off_ state, whereas devices with lower *V*_th_ ​ consistently exhibit the *C*_on_​ state. In contrast, transistor‐mode directly translates threshold voltage and device state variations into output current variation because the drain current is directly affected by *V*_th_ variation. Conversely, capacitor‐mode maintains stable *C*_off_ and *C*_on_ values, demonstrating robustness against both gate voltage variation and device‐to‐device *V*_th_ or state variation. Consequently, reliable binary sensing can be preserved even when the available memory window is reduced, provided that the memory window remains sufficiently larger than the transition region. Figure 4d further supports the reliability of capacitor‐mode sensing by presenting histograms of *C*_on_ and *C*_off_ extracted from 100 randomly selected devices. Both capacitance states exhibit a small standard deviation of 0.022 pF, confirming accurate tuning and clear binary separation across the array. To directly demonstrate the array‐level implications of this robust binary tuning capability, a 48 × 24 down‐sampled Cameraman image encoded in 1‐bit resolution was mapped onto a same‐sized AND‐type array by assigning the binary capacitance states to individual devices. As shown in Figure S2b, the measured capacitance map closely reproduces the target binary image, confirming stable binary state transfer and distinct device‐state separation based on capacitance value.

**FIGURE 5:**
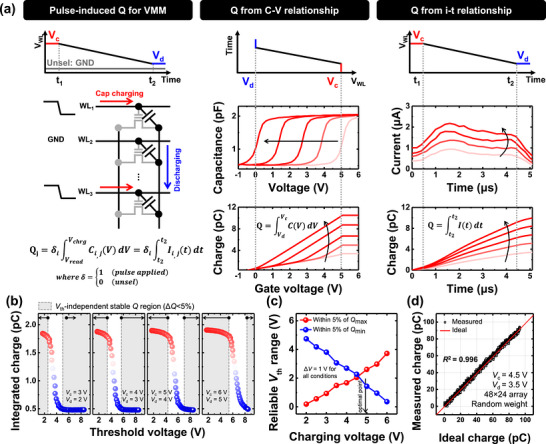
Capacitor‐mode VMM operation and read‐pulse optimization. (a) Schematic illustration of capacitor‐mode VMM operation, where a falling voltage pulse is applied to selected WLs and the resulting discharging currents from individual cells are summed along DLs to perform charge‐domain VMM. (b) Integrated output charge as a function of device *V*_th_ under different charging voltages *V*_c_ while maintaining a constant voltage difference *V*_c_ − *V*_d_ ​ = 1 V. The *V*_th_‐independent stable charge regions (Δ*Q* < 5%) for *Q*_max_​ and *Q*_min_ are highlighted, demonstrating robust binary readout in capacitor‐mode. (c) Extracted *V*_th_ ranges corresponding to stable *Q*_max_​ and *Q*_min_​ (within 5% variation) as a function of *V*_c_, identifying an optimal read‐pulse condition that provides balanced stability for both binary states. (d) Experimental verification of capacitor‐mode VMM operation in a 48 × 24 array using the optimized read‐pulse condition, where the measured output charge exhibits a strong linear correlation with the ideal summed charge.

In addition, VMM in capacitor‐mode is performed by the discharging current through the DLs, as illustrated in Figure 5a. A falling voltage pulse from the charging voltage (*V*_c_​) to the discharging voltage (*V*_d_​) is applied to the selected WLs, while the unselected WLs are grounded. The selected cells discharge according to their capacitance values, and the resulting discharging currents are summed along the DLs to realize the VMM operation. When a falling pulse from *V*_c_ = 5 V to *V*_d_ = 0 V with a fall time of 4 µs is applied, the time‐integrated DL current closely matches the charge determined by the effective device capacitance, confirming accurate capacitor‐mode VMM operations. Here, the 4 µs fall time was selected to reliably capture the transient discharging current using the waveform generator/fast measurement unit (WGFMU) with a 10 ns current sampling resolution. As such, the selected fall time reflects the measurement condition rather than the intrinsic speed limitation of the proposed VMM scheme. Although capacitor‐mode operation requires additional charging and integration processes for charge‐domain sensing compared with transistor‐mode operation, the associated latency overhead can be mitigated through architecture‐level optimizations, including enhanced row and column parallelism [73, 74]. Moreover, because the integration process is performed in parallel across columns, the additional sensing step does not necessarily result in a proportional reduction in throughput. As a result, competitive throughput and latency can be maintained when appropriate architecture‐level optimizations and parallel operation strategies are employed.

Also, to verify that the floating‐SL configuration does not induce memory‐state degradation during repeated read operation, the stability of capacitor‐mode read operation under the floating SL condition was evaluated by repeatedly reading two cells sharing the same SL for up to 10^5^ cycles. As shown in Figure S3, one cell was tuned to the erase state exhibiting *C*_on_ by applying a −18 V, 100 µs erase pulse, while the other cell sharing the same SL was tuned to the program state exhibiting C_off_ using a 16 V, 10 µs program pulse. During the repeated read operation, a falling read pulse from 4.5 to 3.5 V with a fall time of 4 µs was applied to the selected WL, while all unselected WLs were grounded. The transient discharging current from each cell was integrated to extract the charge output. No noticeable degradation in the charge output was observed over 10^5^ repeated read cycles, indicating stable capacitor‐mode read operation under the floating SL condition. This observation further suggests that floating‐SL operation does not induce measurable memory‐state drift during repeated read operations.

To identify an optimal read pulse that enables stable binary‐state output in capacitor‐mode, the voltage difference between *V*_c_​ and *V*_d_​ was fixed at 1 V, while their absolute voltage levels were varied. Under these conditions, the output charge from a single device in the array was measured as a function of device state, as shown in Figure 5b. Specifically, four operating conditions were evaluated by increasing *V*_c_​ from 3 to 6 V in 1 V increments while maintaining *V*_c_​ − *V*_d_ = 1 V. For *V*_c_​ = 3 V and *V*_d_ = 2 V, devices with lower *V*_th_ produced a high integrated charge (*Q*_max_​) of approximately 1.9 pC, remaining within a 5% variation, whereas devices with higher *V*_th_​ consistently yielded a low integrated charge (*Q*_min_​) of approximately 0.5 pC, also within a 5% variation, once outside the transition region. The *V*_th_​‐independent stable charge regions (Δ*Q* < 5%) are highlighted in gray in Figure 5b. Within these regions, the integrated output charge remains nearly constant despite variations in device *V*_th_, enabling robust capacitor‐mode readout that is inherently tolerant to device‐state fluctuations. This behavior confirms the existence of well‐defined operating windows in which reliable binary sensing is maintained irrespective of *V*_th_​ variation.

Furthermore, the same analysis was repeated for higher *V*_c_ values up to 6 V. As the read pulse voltage levels were shifted upward while maintaining *V*_c_ ​ − *V*_d_ = 1 V, the *V*_th_ range over which *Q*_min_​​ remained stable gradually decreased, whereas the stable *V*_th_​ range for *Q*_max_ ​expanded. Figure 5c summarizes these trends by plotting the *V*_th_ ranges corresponding to stable *Q*_min_​​ and *Q*_max_​, each defined within a 5% variation, as a function of *V*_c_​. As a result of these opposing trends, an optimal operating point emerges at an intermediate charging voltage. Specifically, at *V*_c_​ = 4.5 V (with *V*_d_​ = 3.5 V), the stability margins for *Q*_min_​​ and *Q*_max_​ become comparable, providing balanced tolerance for both binary states. At this operating point, variations in device state of up to approximately 2 V in *V*_th_​ result in less than 5% variation in both *Q*_min_​​ and *Q*_max_​, ensuring highly stable binary readout. Using this optimal read pulse condition, Figure 5d evaluates capacitor‐mode VMM operation in a 48 × 24 array. Devices were randomly programmed to either *C*_on_ or *C*_off_ state, and the number of selected WLs gradually increased. The output charge, obtained by integrating the measured DL current, was compared with the calculated summed charge. The measured results closely match the ideal values, yielding an *R* ^2^ of 0.996, which confirms highly accurate VMM operation in capacitor‐mode. This robust charge‐domain operation enables accurate VMM with minimal sensitivity to device variability and analog noise, making capacitor‐mode sensing particularly attractive for energy‐efficient CIM.

## 4 CIM Performance in Dual‐Mode Operations

**FIGURE 6:**
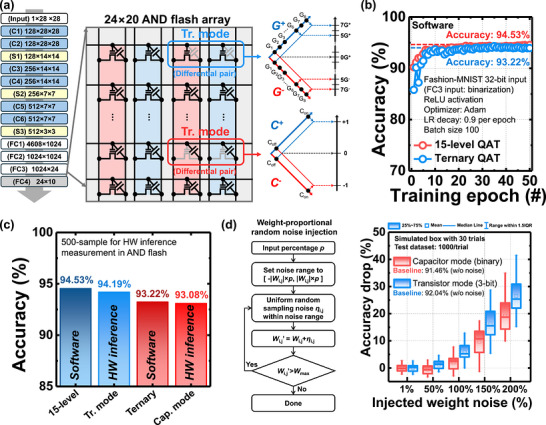
Hardware‐based neural network inference. (a) Architecture of the VGG‐like CNN used for Fashion‐MNIST classification and AND‐type array‐based hardware implementation. The final fully connected layer is mapped onto the fabricated AND‐type flash array. (b) Software‐level QAT results assuming different weight resolutions, where 15‐level quantization is used for transistor‐mode operation and ternary quantization is used for capacitor‐mode operation. (c) Hardware inference accuracy obtained after transferring the trained weights onto the fabricated AND‐type array and performing inference using 500 test samples, showing modest accuracy degradation compared to the software‐level results. (d) Robustness evaluation under device‐state variation, where random noise is injected into the mapped weights and the resulting inference accuracy is summarized as box plots. Capacitor‐mode inference exhibits reduced sensitivity to weight perturbations compared to transistor‐mode inference.

A hardware‐based convolutional neural network (CNN) for Fashion‐MNIST classification was implemented to compare the performance of transistor‐mode and capacitor‐mode operation, as shown in Figure 6a. The final fully connected layer with a size of 24 × 10 was mapped onto a 24 × 20 sub‐array using a differential‐pair scheme. In transistor‐mode, 3‐bit device states were employed to realize 15‐level weight mapping, whereas in capacitor‐mode, 1‐bit device states were used to implement ternary weight mapping. Accordingly, 15‐level quantization‐aware training (QAT) was applied in software for transistor‐mode operation, while ternary QAT was used for capacitor‐mode operation. Figure 6b shows the software‐based training results obtained using QAT under different assumed device state levels. Specifically, 15‐level QAT was applied for transistor‐mode operation, while ternary QAT was used to emulate capacitor‐mode operation. For hardware mapping, the inputs to the third fully connected layer were binarized, and network training was performed for 50 epochs. Under these software‐level conditions, the 15‐level QAT achieved a classification accuracy of 94.53%, whereas the ternary QAT yielded an accuracy of 93.22%. Figure 6c presents the corresponding hardware inference results after transferring the trained weights onto the fabricated array and performing inference using 500 test samples. When transistor‐mode was employed for 15‐level QAT‐based hardware inference, an accuracy of 94.19% was obtained, whereas capacitor‐mode hardware inference based on ternary QAT achieved an accuracy of 93.08%. Compared to the software‐level results, both modes exhibit a modest accuracy degradation after hardware mapping, which can be attributed to non‐idealities such as programming inaccuracies, device‐to‐device variation, and read noise in the physical array. These results indicate that transistor‐mode benefits from higher weight resolution, resulting in higher peak inference accuracy.

To evaluate tolerance to device‐state variation, additional simulations were conducted by injecting random noise into the mapped weights. In transistor‐mode, weights were represented by the drain current corresponding to each multi‐level device state, whereas in capacitor‐mode, weights were represented by the capacitance values corresponding to the binary states *C*_on_ and *C*_off_. For each weight *W*_i,j_​, corresponding to the device located at the *i*‐th WL and *j*‐th DL, a random perturbation was sampled from a uniform distribution within the range [−*p*×*W*_i,j_,+*p*×*W*_i,j_​], where *p* denotes the normalized noise level. The perturbed weights were then used to evaluate inference accuracy. This procedure, illustrated in the flowchart in Figure 6d, was repeated 30 times for each noise condition using a test set of 1,000 samples, and the results are summarized as box plots. Even when the normalized noise level reached 200%, capacitor‐mode inference exhibited a relatively small average accuracy degradation of 19.22% compared to the baseline. In contrast, transistor‐mode inference, which relies on higher‐resolution weight states and is therefore more sensitive to perturbations, showed a larger average accuracy degradation exceeding 26%. These results demonstrate that transistor‐mode enables higher peak accuracy, whereas capacitor‐mode provides superior robustness against device‐state variation, highlighting the complementary advantages of the proposed dual‐mode CIM architecture. This accuracy–robustness trade‐off suggests that capacitor‐mode operation is particularly advantageous under energy‐ or noise‐constrained conditions, while transistor‐mode operation is better suited for accuracy‐critical workloads.

**FIGURE 7:**
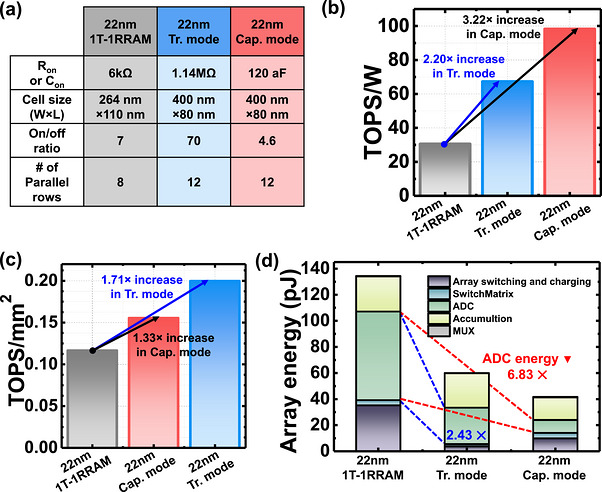
System‐level benchmarking of dual‐mode AND‐flash CIM. (a) Device‐ and system‐level parameters used for array‐level simulations in NeuroSim, comparing the proposed AND‐flash‐based CIM operating in both transistor‐ and capacitor‐modes with a conventional 1T–1RRAM‐based CIM at the 22 nm technology node under a ResNet‐50 workload on the ImageNet dataset. (b) Energy efficiency comparison in terms of throughput per power (TOPS/W), showing improvements for both operating modes relative to the 1T–1RRAM baseline, with capacitor‐mode achieving the highest energy efficiency. (c) Area efficiency comparison using throughput density (TOPS/mm^2^), where transistor–mode achieves higher throughput density due to its higher computational speed, while capacitor‐mode provides moderate improvement over the baseline. (d) Energy consumption of the proposed dual‐mode CIM architecture, showing substantial energy reduction in both operating modes compared to the 1T–1RRAM baseline, with a more significant reduction in capacitor‐mode.

Additional system‐level simulations were conducted using the CIM benchmarking framework NeuroSim. Specifically, system‐level power consumption and chip area were estimated by accounting for the power and area overheads of peripheral circuits and array operations. The proposed AND‐flash‐based CIM operating in transistor‐mode and capacitor‐mode was compared with a conventional 1T–1RRAM‐based CIM array at the 22 nm technology node under a ResNet‐50 workload on the ImageNet dataset [75, 76]. The measured device characteristics of the AND‐flash‐based CIM in both transistor‐ and capacitor‐modes were appropriately scaled to the 22 nm technology node and incorporated into the NeuroSim framework. The device‐ and system‐level parameters used in the simulations are summarized in Figure 7a. To evaluate energy efficiency, the throughput‐per‐power metric (TOPS/W) was extracted and compared with that of the 1T–1RRAM‐based CIM, as shown in Figure 7b. Transistor‐mode operation achieves a 2.20× improvement in TOPS/W, while capacitor‐mode, benefiting from lower energy consumption per operation, provides a larger improvement of 3.22×. These results indicate that capacitor‐mode is particularly well suited for energy‐constrained and low‐power applications.

Area efficiency was further evaluated using the throughput density metric (TOPS/mm^2^), as summarized in Figure 7c. Compared to the 1T–1RRAM baseline, capacitor‐mode achieves a 1.33× improvement in TOPS/mm^2^, whereas transistor‐mode exhibits a higher enhancement of 1.71×. Here, the identical physical array area refers specifically to the memory‐cell array region based on the same AND‐type flash cell and TANOS stack, rather than the total chip‐level area including peripheral circuits. In practical implementations, capacitor‐mode sensing may require additional peripheral circuits, such as charge/capacitance readout circuits, reference components, and SL isolation switches for floating‐SL operation. Therefore, the reported area efficiency reflects the peripheral configuration assumed in the NeuroSim evaluation, while practical implementations may exhibit different area‐efficiency trade‐offs depending on the peripheral design. Under the assumed readout configuration, transistor‐mode shows higher TOPS/mm^2^ than capacitor‐mode.

Importantly, as illustrated in Figure 7d, the analog‐to‐digital converter (ADC) energy consumption is significantly reduced in both modes, with a 6.83× reduction in capacitor‐mode and a 2.43× reduction in transistor‐mode compared to the 1T–1RRAM baseline. This substantial reduction in ADC energy contributes directly to the improved system‐level energy efficiency of the proposed architecture. Taken together, these results demonstrate that capacitor‐mode, with its superior energy efficiency and reduced ADC overhead, is well suited for ultra‐low‐power operation, whereas transistor‐mode provides higher throughput and accuracy for performance‐critical workloads. These complementary characteristics enable workload‐dependent mode selection within the same hardware platform. For example, transistor‐mode operation can be employed for accuracy‐critical tasks, whereas capacitor‐mode operation can be utilized under energy‐constrained conditions where robustness and low‐power operation are prioritized. Such mode selection can be achieved without modifying the memory‐cell structure, requiring only the corresponding biasing and readout configuration. This flexibility highlights the practicality of the proposed dual‐mode CIM architecture and enables energy‐adaptive operation according to workload requirements. Since both modes employ identical program and erase schemes, the write operation and its associated energy consumption remain unchanged regardless of the selected operating mode. Consequently, the benefits of the proposed dual‐mode architecture primarily arise from the read operation and become most pronounced in read‐dominant inference workloads employing off‐chip training, where the programmed weights are reused over a large number of VMM operations.

## 5 Conclusion

In this work, we demonstrated a dual‐mode CIM architecture based on an AND‐type flash array that supports both transistor‐mode and capacitor‐mode operation within a unified device structure. By selectively exploiting current‐based sensing for high‐precision computation and charge‐based sensing for ultra‐low‐power operation, the proposed architecture enables distinct operating characteristics within the same memory‐cell and array structure, with the operating mode determined by the corresponding bias and readout conditions. At the device and array levels, transistor‐mode provides fine multilevel weight representation and accurate analog VMM, while capacitor‐mode offers binary yet highly robust charge‐domain operation with near‐zero static power consumption. Hardware‐based neural network inference confirms that transistor‐mode achieves higher peak accuracy owing to its higher weight resolution, whereas capacitor‐mode maintains competitive accuracy with significantly improved tolerance to device‐state variation and noise. System‐level benchmarking using NeuroSim further reveals that capacitor‐mode delivers superior energy efficiency and substantially reduced ADC overhead, while transistor‐mode achieves higher throughput density. These complementary characteristics allow the same hardware platform to operate efficiently across a wide range of workloads, from energy‐constrained edge inference to performance‐critical computing tasks. Overall, the proposed dual‐mode AND‐flash CIM architecture provides a practical and scalable approach to workload‐adaptive in‐memory computing, enabling flexible trade‐offs between accuracy, throughput, and energy efficiency. This work highlights how a unified flash memory platform can enable adaptive computing paradigms that dynamically balance accuracy, robustness, and energy efficiency within a single hardware platform.

## 6 Experimental Section

### 6.1 Electrical Measurements

The fabricated 48 × 24 AND‐type CTF array was electrically characterized using a probe station equipped with a 96‐channel probe card. The probe card provided access to 48 WLs, 24 SLs, and 24 DLs, which were connected to an E5250A low‐leakage switching matrix. The switching matrix was interfaced with a semiconductor parameter analyzer (B1500A) equipped with source measure units (SMUs), a semiconductor pulse generator unit (SPGU), a multi‐frequency capacitance measurement unit (MFCMU), and a WGFMU. The capacitance characteristics were measured at 10 kHz using the MFCMU of the B1500 parameter analyzer, where 10 kHz was selected as a standard frequency for *C–V* characterization. The E5250A switching matrix enabled flexible routing among the 96 channels, allowing selective connection of individual WLs, SLs, and DLs to the appropriate measurement units. The entire measurement system was controlled by a custom‐developed C++ program, with both the semiconductor parameter analyzer and the switching matrix connected via a general‐purpose interface bus (GPIB). Using this setup, the electrical characteristics of the AND‐type CTF array were systematically evaluated in both transistor‐mode and capacitor‐mode.

### 6.2 Neural Network Architecture and Hardware Mapping

A VGG‐like convolutional neural network was employed for AND‐type array‐based hardware implementation using the Fashion‐MNIST dataset consisting of 1 × 28 × 28 grayscale images. The network is composed of repeated convolutional blocks, each consisting of 3 × 3 convolution layers followed by batch normalization and ReLU activation, interleaved with 2 × 2 max‐pooling layers. Specifically, the first convolutional block (C1, C2) processes feature maps with 128 channels at a spatial resolution of 28 × 28, followed by max pooling to down‐sample the feature maps to 128 × 14 × 14. The second block (C3, C4) increases the channel dimension to 256 and operates at a 14 × 14 resolution, after which max pooling reduces the feature maps to 256 × 7 × 7. Subsequently, the third block (C5, C6) processes 512‐channel feature maps, which are further reduced to a spatial resolution of 3 × 3. After flattening, the network includes four fully connected layers (FC1‐FC4) with dimensions of 4608 × 1024, 1024 × 1024, 1024 × 24, and 24 × 10, respectively. Batch normalization and ReLU activation are applied after each fully connected layer except for the final output layer. To account for hardware implementation constraints, deterministic weight quantization was applied to all convolutional and fully connected layers during both training and inference, using 3‐bit precision for transistor‐mode operation and 1‐bit precision for capacitor‐mode operation. In addition, the input vector to the final classification layer (FC4) was binarized using a sign‐based bipolar representation. The network was trained using the cross‐entropy loss function for 100 epochs with the Adam optimizer and an initial learning rate of 0.01. The learning rate decayed by a factor of 0.9 at each epoch. For inference, the final fully connected layer with dimensions of 24 × 10 was physically mapped onto the AND‐type CTF array. Weight transfer was performed using drain current levels for 3‐bit weights in transistor‐mode and device capacitance states for 1‐bit weights in capacitor‐mode.

## Conflicts of Interest

The authors declare no conflicts of interest.

## Supporting information




**Supporting file**: advs76619‐sup‐0001‐SuppMat.docx

## Data Availability

The data that support the findings of this study are available from the corresponding author upon reasonable request.
