# Supplementary material for: A Unified Flash Memory Platform for Mode‐Adaptive and Robust AI Computation
Source: Adv Sci (Weinh). 2026 Jul 21;13(56):e76619. doi: 10.1002/advs.76619 (PMC13387032; doi:10.1002/advs.76619)
Supplement: Supplementary file 1 [file ADVS-13-e76619-s001.docx]

Supporting Information

**A Unified Flash Memory Platform for Mode-Adaptive and Robust AI Computation**

Dayeon Yu^1^, Hwiho Hwang^1^, Byeongchan Oh^2^, Junmo Lee^3^, Shimeng Yu^3*^, Tae-Hyeon Kim^2*^, and Hyungjin Kim^1*^

^1^Division of Materials Science and Engineering and Department of Semiconductor Engineering, Hanyang University, Seoul 04763, Korea

^2^Department of Semiconductor Engineering, Seoul National University of Science and Technology, Seoul, Korea

^3^School of Electrical and Computer Engineering, Georgia Institute of Technology, Atlanta, GA, 30332, USA

*Corresponding author email: [shimeng.yu@ece.gatech.edu](mailto:shimeng.yu@ece.gatech.edu), [teahyeon@seoultech.ac.kr](mailto:teahyeon@seoultech.ac.kr) and [hkim12@hanyang.ac.kr](mailto:hkim12@hanyang.ac.kr)

**Fig. S1. Program, erase, and inhibit characteristics of the AND-type flash array.** (a) Summary of the bias conditions applied to the selected and unselected WLs, DLs, and SLs during program, erase, and read operations in both transistor mode and capacitor mode. A *V*/2 bias scheme is employed to suppress undesired program and erase operations in unselected cells. (b) *V*_th_ shifts of the selected cell and the surrounding eight half-selected neighboring cells during ISPP with a pulse width of 10 μs, demonstrating effective program inhibit behavior. (c) *V*_th_ shifts of the selected cell and neighboring half-selected cells during ISPE with a pulse width of 100 μs, confirming reliable erase inhibit operation within the AND-type array.

**Fig. S2. Array-level weight transfer demonstrations in transistor mode and capacitor mode.** (a) Array-level weight transfer in transistor mode using a 48 × 24 down-sampled Cameraman image encoded in 3-bit resolution. Each device in the AND-type flash array is programmed to one of eight discrete drain current levels according to the target image, and the measured drain current map is shown, demonstrating accurate and spatially uniform multi-level weight transfer. (b) Array-level binary weight transfer in capacitor mode using the same 48 × 24 down-sampled Cameraman image encoded in 1-bit resolution. The binary information is mapped onto the device capacitance states (*C*_on_ and *C*_off_), and the measured capacitance map closely reproduces the target image, confirming stable binary state transfer and clear device-state discrimination based on capacitance magnitude.

**Fig. S3. Stability of capacitor-mode read operation under floating-SL condition.** Charge output extracted during repeated capacitor-mode read operation with the SL left floating. Two cells sharing the same SL were tuned to the program and erase states by applying a 16 V, 10 μs program pulse and −18 V, 100 μs erase pulses, respectively. During each read cycle, a falling pulse from 4.5 V to 3.5 V with a fall time of 4 μs was applied, and the transient discharging current was integrated to obtain the charge output. No noticeable degradation was observed over 10^5^ read cycles, confirming stable capacitor-mode read operation without measurable memory-state drift under the floating-SL condition.
